# The osteogenic commitment of CD271+CD56+ bone marrow stromal cells (BMSCs) in osteoarthritic femoral head bone

**DOI:** 10.1038/s41598-020-67998-0

**Published:** 2020-07-07

**Authors:** Dragos C. Ilas, Thomas G. Baboolal, Sarah M. Churchman, William G. Jones, Peter V. Giannoudis, Hans-Jörg Bühring, Dennis McGonagle, Elena Jones

**Affiliations:** 1 Leeds Institute of Rheumatic and Musculoskeletal Medicine, The University of Leeds, Leeds, UK; 2 NIHR Leeds Musculoskeletal Biomedical Research Unit, Leeds, UK; 3 Institute of Medical and Biological Engineering, University of Leeds, Leeds, UK; 4 Department of Internal Medicine II, University of Tübingen, Tübingen, Germany

**Keywords:** Cell biology, Stem cells, Rheumatology, Osteoarthritis

## Abstract

Osteoarthritis (OA), the most common joint disorder, is characterised by progressive structural changes in both the cartilage and the underlying subchondral bone. In late disease stages, subchondral bone sclerosis has been linked to heightened osteogenic commitment of bone marrow stromal cells (BMSCs). This study utilised cell sorting and immunohistochemistry to identify a phenotypically-distinct, osteogenically-committed BMSC subset in human OA trabecular bone. Femoral head trabecular bone tissue digests were sorted into CD45-CD271+CD56+CD146-, CD45-CD271+CD56-CD146+ and CD45-CD271+CD56-CD146-(termed double-negative, DN) subsets, and CD45+CD271-hematopoietic-lineage cells served as control. Compared to the CD146+ subset, the CD56+ subset possessed a lower-level expression of adipocyte-associated genes and significantly over 100-fold higher-level expression of many osteoblast-related genes including osteopontin and osteocalcin, whilst the DN subset presented a transcriptionally ‘intermediate’ BMSC population. All subsets were tri-potential following culture-expansion and were present in control non-OA trabecular bone. However, while in non-OA bone CD56+ cells only localised on the bone surface, in OA bone they were additionally present in the areas of new bone formation rich in osteoblasts and newly-embedded osteocytes. In summary, this study reveals a distinct osteogenically-committed CD271+CD56+ BMSC subset and implicates it in subchondral bone sclerosis in hip OA. CD271+CD56+ subset may represent a future therapeutic target for OA and other bone-associated pathologies.

## Introduction

Osteoarthritis (OA) is now recognised as a disease of the whole joint characterised by pathological changes to cartilage, subchondral bone and the synovium^1,2^. While the site of initiation is still unclear, subchondral bone changes are an important feature in OA pathophysiology^3^. Given the high numbers of bone marrow stromal cells (BMSCs) in the subchondral bone^4–6^, their homeostatic role in health, as well as their potential contribution to the endogenous repair mechanisms in OA is currently an area of considerable interest. Recent studies in OA animal models^7^ and in human OA^8,9^ point towards an altered commitment of BMSCs in OA progression. BMSCs have been long described as a heterogeneous population of cells^10,11^ however, no specific markers capable of identifying and segregating differently-committed BMSC subsets in healthy or OA-affected bone have so far been found.

Currently, CD271 is considered as one of the characteristic markers for native human BMSC^12–15^. We have previously shown an accumulation of CD271+ BMSCs in the damaged femoral head areas of hip OA patients marked by the presence of bone marrow lesions^8^, which are also known to be associated with cartilage loss and bone sclerosis^16^. More recently, we have provided further evidence for increased osteogenic activity in OA subchondral bone, implicating both BMSCs and their terminally differentiated progeny osteocytes in hip OA microstructural changes^9^. Specifically, the CD271+ BMSCs were found to accumulate in areas of bone sclerosis and to reside in direct proximity to osteoblasts and immature osteocytes. Additionally, their gene expression profile indicated a predilection for bone formation as evident by the elevated levels of numerous osteogenic-lineage molecules as well as the early osteocyte marker, E11^9^. Therefore, we hypothesised that a distinct osteogenically-committed BMSC subset could be found in human trabecular bone and be elevated in OA femoral head bone.

In a pioneering study by Tormin et al., healthy CD271+ BMSCs were segregated into bone-lining and perivascular subsets, the latter marked by the presence of CD146, also known as melanoma-cell adhesion molecule^17^. CD146 has been earlier proposed as a marker of perivascular progenitors in many tissues, which allow these cells to gain easy access to wider tissue area if repair is needed^18,19^. The phenotypic and functional identity of the bone-lining subset of CD271+ BMSCs remained however less explored^17^. CD56, also known as neural cell adhesion molecule, has been previously shown to be expressed on a small subset of CD271+ BMSCs aspirated from healthy bone marrow^20,21^. More recently, CD56 was documented to co-localise with CD271 on the surface of both healthy and OA trabecular bone, but not be present in the perivascular regions^22^. At this bone-lining location, CD56 expression was independently documented not only in a flat thin cell layer of cells^23^, but also in active osteoblasts that coincided with their deposition of collagen I and alkaline phosphatase activity^24^. In accumulation, this indicated that CD56 could be acquired by BMSCs as they progressed along the osteogenic lineage, and could, therefore, be a potential marker of osteogenically-committed BMSCs. Given the accelerated bone remodelling and sclerosis that is characteristic of OA, it could be predicted that such a BMSC subset might be increased at this location.

The aims of this study were therefore to investigate the presence of CD271+CD56+ BMSCs in OA bone, and to test their gene expression for further elucidation of their potential contribution to OA associated bone changes.

## Results

### Gene expression profile of CD271+ BMSC subsets in OA femoral head trabecular bone

**Figure 1:**
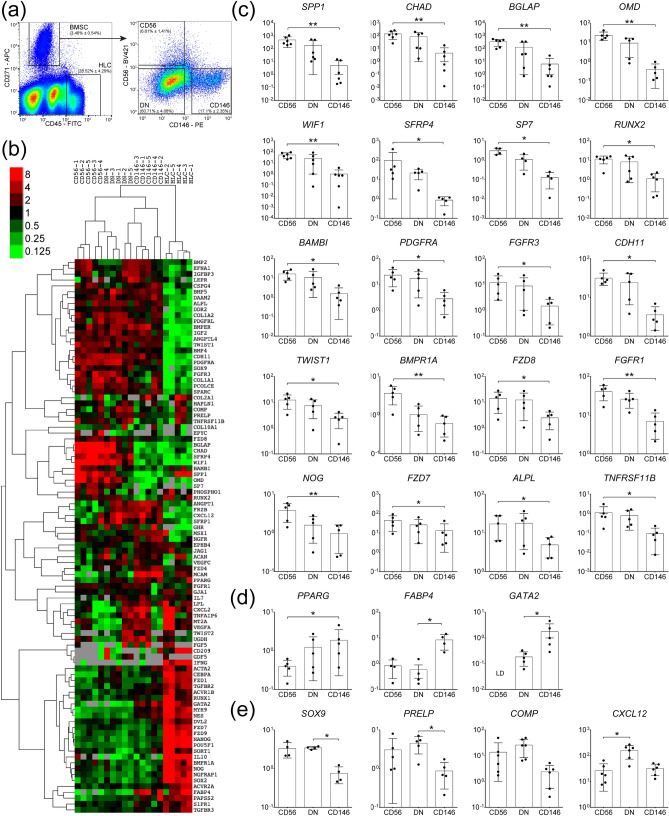
Gene expression profile of CD56+ BMSC subset in OA femoral head trabecular bone. (**a**) Cell sorting strategy based on the identification of CD45-CD271+ BMSCs and negative control CD45+CD271- HLCs followed by the separation of BMSCs into the three distinct subsets: CD56+ (CD45-CD271+CD56+), CD146+ (CD45-CD271+CD146+) and DN (CD45-CD271+CD56-CD146-) (proportion of each subpopulation is presented as mean ± SD, n = 23 donors). (b) Dendrogram showing hierarchical cluster analysis of the BMSCs subsets and HLCs gene expression from OA trabecular bone; the colour scale reflects the log transformed fold differences relative to *HPRT1*; grey—missing data (below detection). (**c**) Osteogenesis-related genes expressed at significantly higher levels in CD56+ subset compared to CD146+ subset. (**d**) Genes expressed at lower levels in CD56+ subset compared to CD146+ subset. (**e**) Chondrogenesis associated genes and *CXCL12*. Data are represented as bar graphs (means) with standard deviations (error bars) for donor-matched samples and gene expression values are relative to *HPRT1* on a log scale. LD: low detection; * < 0.05, ** < 0.01, Friedman test for the donor matched samples corrected with the Bonferroni–Dunn multiple-group comparison. While n = 6 donor samples were analysed, only n = 5 had complete datasets for all the sorted subsets to be presented in the dendrogram (**b**). However, where present the genes with 6 matching data points are shown for full transparency of acquired data (**c**–**e**).

To investigate the existence of phenotypically distinct BMSC subsets in OA trabecular bone, enzymatically extracted cells were processed for cell sorting and the BMSCs were gated based on the CD45-CD271+ phenotype while the CD45+CD271- hematopoietic-lineage cells (HLCs) were used as a negative non-BMSC control, as previously described^25–27^ (Fig. 1a). In all samples, non-overlapping subsets of cells CD271+CD56+ (abbreviated CD56+) and CD271+CD146+ cells (abbreviated CD146+) were found while the majority of the CD45-CD271+ BMSC population lacked both CD56 and CD146 and was termed the double negative (DN) subset (Fig. 1a).

All three CD271+ BMSC subsets were next sorted from OA femoral head trabecular bone digests for downstream gene expression analysis. Sorting gates were set as shown on Fig. 1a and a panel of 96 genes was used, summarised in Supplementary Table 1, to include transcription factors (TFs) and mature proteins involved in BMSC osteogenic, adipogenic and chondrogenic differentiation, as well as selected molecules shown to be highly expressed in in vivo CD45-CD271+ BMSCs from previous microarray and gene expression studies^17,26,28,29^. The gene expression results were subjected to hierarchical clustering^26^, and showed a clear separation of all three BMSC subsets away from the HLCs, as expected (Fig. 1b). Furthermore, within the CD271+ BMSC population, CD56+ and CD146+ subsets clustered away from each other whilst the DN subset was positioned in-between, potentially suggesting its transitional nature.

The statistical analysis of individual genes expression in the three subsets revealed that out of 94 tested genes, 20 of them were expressed > twofold higher in the CD56+ subset compared to CD146+ subset (Table 1). The most differentially expressed genes (showing > 100 fold differences in their expression) were the genes encoding two mature bone proteins osteopontin (*SPP1*, 508-fold, *p* = 0.0016) and osteocalcin (*BGLAP*, 163-fold, *p* = 0.0045), the latter also present on active osteoblasts^30^, as well as chondroadherin (*CHAD*)*,* which is involved in both osteogenesis and chondrogenesis^31^ (221-fold, *p* = 0.0045) (Fig. 1c). The level of *OMD*, encoding osteomodulin, an extracellular matrix protein with important roles in osteoblasts formation and mineralisation^32^ was 86-fold higher (*p* = 0.0047) in the CD56+ compared to CD146+ subset (Fig. 1c).

**Table 1:** Genes differentially expressed between CD56+ and CD146+ BMSC subsets from OA femoral head bone.

Gene symbol	Gene name	Fold difference	*p* value*
*SPP1*	Secreted phosphoprotein 1 (osteopontin)	508.62	0.0045
*CHAD*	Chondroadherin	221.22	0.0045
*BGLAP*	Bone gamma-carboxyglutamate protein (osteocalcin)	163.11	0.0016
*OMD*	Osteomodulin	86.02	0.0047
*WIF1*	WNT inhibitory factor 1	80.85	0.0045
*SFRP4*	Secreted frizzled-related protein 4	40.36	0.0133
*SP7*	Sp7 transcription factor	25.78	0.0140
*TNFRSF11B*	Tumour necrosis factor receptor superfamily, member 11b (osteoprotegerin)	15.53	0.0342
*RUNX2*	Runt-related transcription factor 2	13.57	0.0117
*BAMBI*	BMP and activin membrane-bound inhibitor homolog	11.75	0.0133
*PDGFRA*	Platelet-derived growth factor receptor, alpha polypeptide	10.31	0.0342
*FGFR3*	Fibroblast growth factor receptor 3	8.12	0.0047
*CDH11*	Cadherin 11, type 2, OB-cadherin	6.93	0.0342
*TWIST1*	Twist homolog 1	6.91	0.0133
*BMPR1A*	Bone morphogenetic protein receptor, type IA	6.20	0.0047
*FZD8*	Frizzled homolog 8	6.01	0.0342
*FGFR1*	Fibroblast growth factor receptor 1	5.07	0.0047
*NOG*	Noggin	3.82	0.0047
*FZD7*	Frizzled homolog 7	3.62	0.0133
*ALPL*	Alkaline phosphatase	2.78	0.0342
*PPARG*	Peroxisome proliferator-activated receptor gamma	0.38	0.0133
*GATA2*	GATA binding protein 2	BD in CD56	NA

* Friedman test for the donor matched samples with the Bonferroni–Dunn correction for multiple-group comparisons. *BD* below detection, *NA* not available.

The complete list of differentially expressed molecules between CD56+ and CD146+ subsets is shown in Table 1 and also includes osteogenic TF *SP7* (osterix) and many molecules belonging to Wnt and BMP signalling pathways. Of note, for the majority of these differentially expressed genes, their average expression in the DN subset was intermediate between the CD56+ and CD146+ subsets (Fig. 1c).

The only gene that was found significantly lower in CD56+ subset compared to CD146+ cells was TF *PPARγ* commonly associated with adipogenesis (2.6-fold, *p* = 0.0133) (Fig. 1d). Interestingly, the expression of *FABP4*, encoding a mature adipocyte protein (fatty acid-binding protein 4) was lower in both CD56+ and DN cells compared to CD146+ cells (12- and 16-fold, respectively) however, the differences only reached statistical significance for the DN subset (*p* = 0.0140). Finally, another gene that was significantly lower in DN subset compared to CD146+ subset (sixfold, *p* = 0.0159) (Fig. 1d) and importantly below detection in CD56+ subset, was *GATA2*, a TF initially described as specific for haematopoietic stem cells^33^, but subsequently shown to have a role in the differentiation of BMSCs^34^.

While some genes associated with BMSC chondrogenesis (*SOX9*, *PRELP* encoding prolargin and *COMP* encoding cartilage oligomeric matrix protein) showed higher expression levels in both CD56+ and DN subsets compared to CD146+ subset, they didn’t show a trend for higher-level expression in the CD56+ subset compared to the DN subset (Fig. 1e). Finally, *CXCL12*, encoding a well-known stromal support chemokine SDF-1^35^, showed markedly higher expression in DN cells compared to both CD56+ (sevenfold, *p* = 0.0133) and CD146+ (fourfold) subsets (Fig. 1e), although the latter was not statistically significant.

Taken together, this gene expression analysis revealed distinct transcriptional profiles of CD56+, DN and CD146+ BMSC subsets in OA femoral head bone, and an increased osteochondral commitment from the CD146+ subset to DN and CD56+ subsets, followed by an increased osteogenic commitment from the DN to the CD56+ subset.

### Multipotentiality and distinct morphology of the CD56+ BMSC subset in OA femoral head trabecular bone

**Figure 2:**
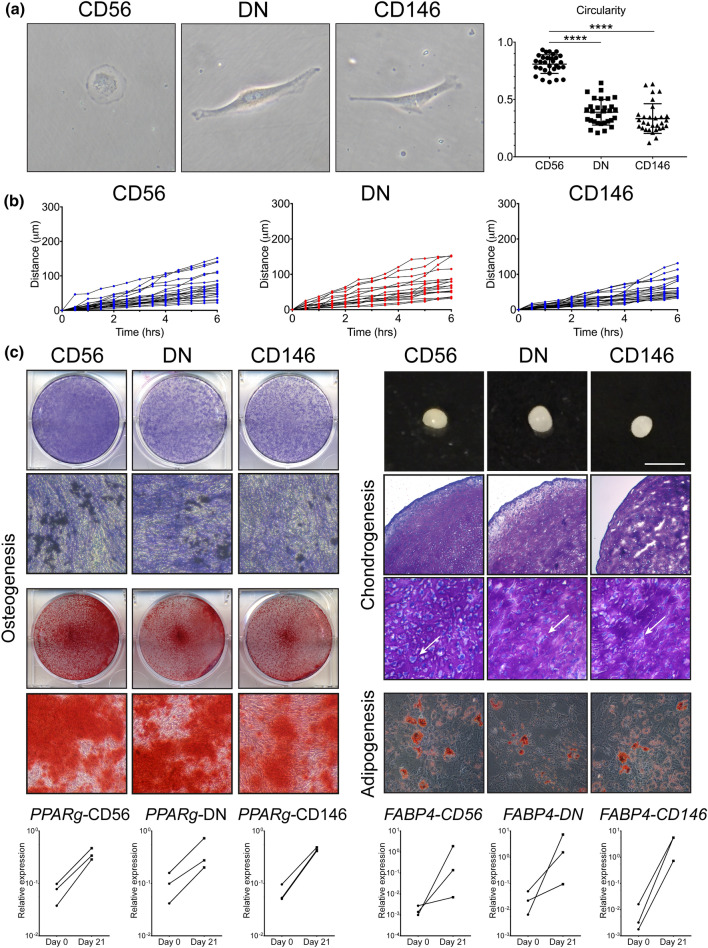
Morphological and migratory characteristics of BMSC subsets in early culture and their multipotentiality following standard culture-expansion. (**a**) Morphological analysis of the CD56+, DN and CD146+ BMSC subsets 24 h after FACS purification. Left: representative cells, right: graph showing the cell circularity calculated using ImageJ (a total of n = 30 cells in each subset from n = 5 OA donors). **** < 0.0001, Kruskal–Wallis test. (**b**) Cell motility calculated based on the total distance (μm) travelled by individual cells over the course of 6 h, each line representing a single cell over time. (**c**) Representative images showing tri-lineage differentiation potentials of CD56+, DN and CD146+ BMSC subsets, all assays were performed in triplicate wells/pellets from each of n = 3 OA donors. Osteogenic differentiation was assessed by ALP staining (blue) on day 14 post-osteogenic induction with visible mineralising nodules (dark spots) and by alizarin red staining (red) on day 21. Chondrogenic differentiation was assessed by generating chondrogenic pellets (bar indicating 2 mm) and toluidine blue staining of glycosaminoglycans in the pellet cultures (both on day 21). Higher magnification images show substantial extracellular matrix deposition and bona fide chondrocytes lying in lacunae within the pellets (indicated by arrows). Adipogenic differentiation was visualised by oil-red-O staining of lipid vacuoles and by transcriptional analysis of adipogenic transcripts *PPARG* and *FABP4* on day 21 post-induction; the data are presented as donor-matched connecting lines in n = 3 donors. Images were taken using Epson scanner for low magnification (ALP- and AR-stained plates and whole chondrogenic pellets) and using Nikon camera attached to Nikon microscope for microphotographs (osteogenesis: × 20, chondrogenesis: × 20 and × 40 (toluidine blue sections), adipogenesis: × 20.

We next investigated the morphological characteristics and the multipotentiality of the purified BMSC subsets in vitro. Cells were sorted as indicated on Fig. 1a, and placed in BMSC culture media for the analysis of their morphology, motility and formation of tripotential cultures (Fig. 2).

Firstly, a notable morphological difference was observed between the CD56+ subset and the other two subsets (CD146+ and DN) following their attachment to plastic (1 day after sorting). The CD56+ subset displayed a circular shape, while the DN and CD146+ cells displayed a spindle shape cell morphology (Fig. 2a). This was assumed to be related to inherent cell motility and confirmed by measurements of cell circularity, where 0 represented a straight line and 1 represented a perfect circle^36^. The average circularity of the cells from the CD56+ subset was close to 1 (median 0.823, range 0.652–0.933), while the DN and CD146+ cells were significantly more elongated (median 0.398, range 0.209–0.644 and median 0.301, range 0.122–0.635, respectively). However, these distinct features were not maintained in culture, and by day 7 all the subsets adopted a spindle-shape morphology and displayed similar heterogeneity in their rates of motility (Fig. 2b).

Upon reaching the first passage, cells from all the three subsets were subjected to osteogenic, adipogenic and chondrogenic induction in standard trilineage differentiation assays^37^ (Fig. 2c). Culture-expanded cells from all three subsets responded well to osteogenic, adipogenic and chondrogenic induction, and no particular differences in their ALP activity or calcium deposition were noted on day 21 post-osteogenic induction (Fig. 2c). A slight trend for a more adipogenic response was noted in the CD146+ subset compared to the other two subsets based on day-21 Nile red/DAPI fluorescence quantification (Supplementary Fig. 1a). This was also observed by qPCR by measuring transcripts specific to adipogenic lineage cells, *PPARγ* and *FABP4* (Fig. 2c, bottom panel). While all subsets responded to adipogenic induction by increasing the levels of these transcripts, the fold-changes between Day 0 and Day 21 post-induction were notably higher in the CD146 subset, especially for *FABP4* (average 2000-fold) while in CD56+ and DN subsets its levels were elevated on average 100-fold and 70-fold, respectively (Fig. 2c, bottom panel). The diameter measurements of the chondrogenic pellets on day 21 post-chondrogenic induction (Fig. 2c) showed a lower diameter of the CD146+ subset, but no significant differences were observed between the BMSC subsets in the sGAG levels (Supplementary Fig. 1a). Similar day-21 levels of selected lineage-specific transcripts were found by qPCR for osteogenesis while chondrogenesis-related transcripts were notably below detection in the CD146+ subset and the highest in the CD56+ subset (Supplementary Fig. 1b).

Altogether, these data showed that all three sorted subsets were multipotential following adherence to plastic and standard culture, confirming their BMSC identity. However, their native gene expression and morphological traits were not faithfully preserved in culture, confirming previous reports^26,28^ and highlighting the limitation of using cultured BMSCs for the analysis of BMSC heterogeneity in vivo.

### The topography and quantification of CD56+ cells in OA and control trabecular bone

**Figure 3:**
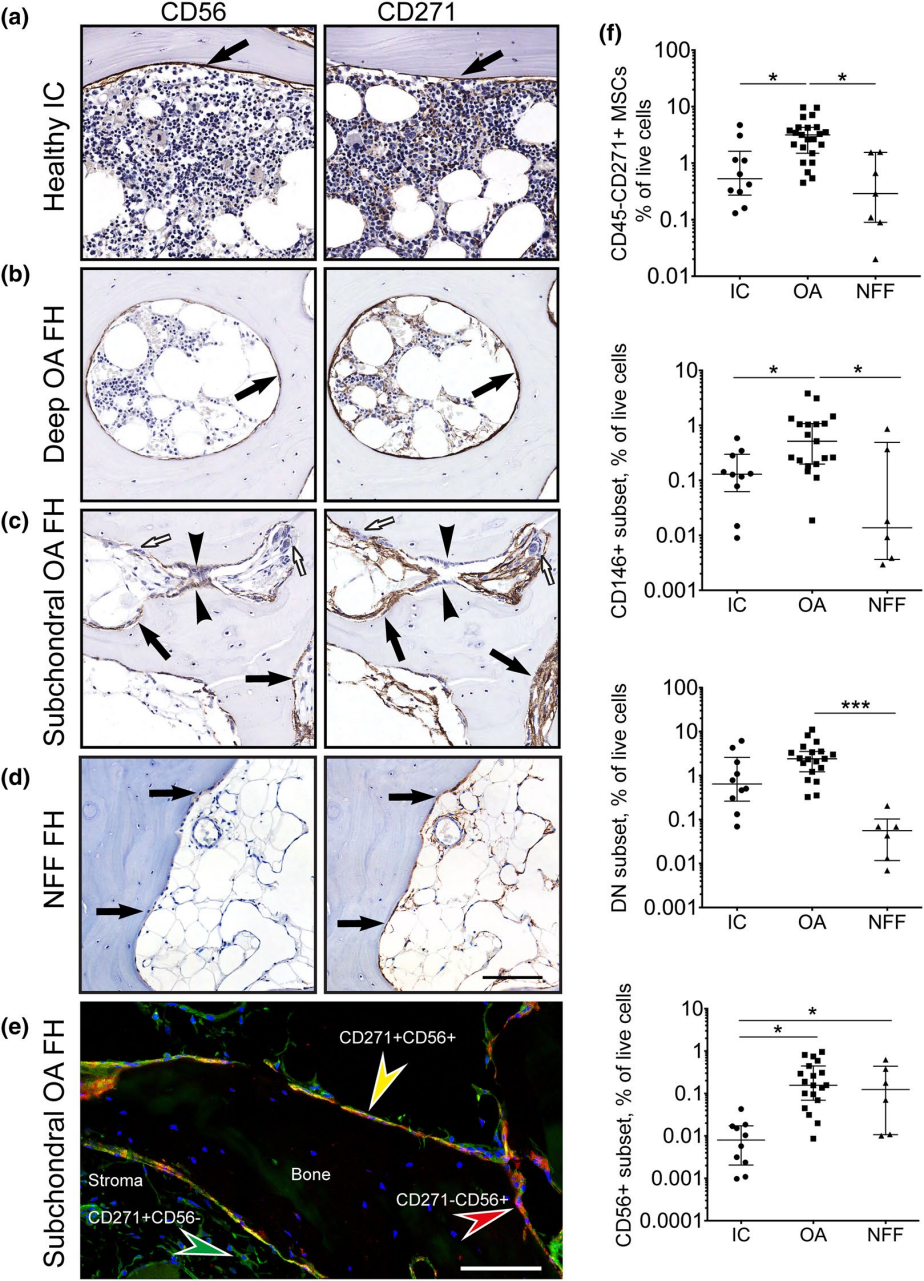
The topography (**a**–**e**) and quantification (**f**) of CD271+CD56+ BMSC subset in osteoarthritic and control trabecular bone. Representative adjacent sections (5 μm) stained with antibodies against CD56 and CD271. (**a**) Heathy iliac crest, (**b**) Deep area of trabecular bone in OA femoral head, (**c**) subchondral area with active bone remodelling area of OA femoral head and (**d**) subchondral area of NFF femoral head. Arrows: bone lining location of CD56+ and CD271+ cells, arrow heads: osteoblasts, white arrows: osteoclasts. Scale bar 100 μm. (**e**) immunofluorescence double-staining of subchondral area of OA femoral head for CD271 (green) and CD56 (red) indicating their co-expression on the cells at the bone surface (yellow); blue: nuclear staining using DAPI; Scale bar 50 μm. (**f**) Quantification of native CD45-CD271+ BMSCs extracted from trabecular bone of OA donors (N = 23) in comparison to healthy IC (N = 11) and NFF (N = 6) trabecular bone. Graphs presented as dot plots show the percentages of CD45-CD271+ BMSCs in total live cells as well as the percentages of each of the CD271+ BMSC subset in total live cells. Each dot represents one donor. * < 0.05, *** < 0.001, Kruskal–Wallis test.

The topographical distribution of CD271- and CD56-expressing cells was next investigated in OA femoral head trabecular bone and control iliac crest (IC) trabecular bone and femoral head trabecular bone from the neck of femur fracture (NFF) patients (Fig. 3). In control IC bone, the expression of CD56 was limited to a thin layer of cells lining the trabecular surface in a similar location as that of CD271 expressing cells (Fig. 3a). In femoral head bone from OA patients, two patterns of CD56 expression were found depending on the proximity of bone to damaged cartilage areas. In deep femoral head areas not visibly affected by OA, CD56+ cells formed a single layer of bone-lining cells, similar to control bone (Fig. 3b, left panel). However, in the subchondral areas showing active bone remodelling signs, with a characteristic presence of osteoblasts and osteoclasts, CD56 was present on multiple layers of cells proximal to bone, as well as on some osteoblasts (Fig. 3c, left panel). In NFF bone, CD56 was limited to the cells present on the bone surface (Fig. 3d, left panel). Co-localisation of CD271 and CD56 on bone-lining cells, as well as the presence of single marker-positive cells in OA subchondral bone was confirmed by dual staining immunofluorescence (Fig. 3e). In all samples, CD271+ cells were more numerous compared to CD56+ cells and in addition to the cells lining the bone, included the stromal cells present inside the bone cavities and around blood vessels (Fig. 3a–d, right panels and e).

The proportions of CD271+CD56+ cells, as well as other BMSC subsets, were next quantified in bone enzymatic digests using flow cytometry (Fig. 3f). In relation to total live cells, the whole fraction of CD45-CD271+ BMSCs was the highest in OA bone (6- and 11-fold higher compared to IC and NFF bone, *p* = 0.0168 and *p* = 0.0021, respectively), with the same pattern seen for the CD146+ and DN BMSC subsets (Fig. 3f). The CD56+ subset was similar in proportion in OA and NFF enzymatic digests, and higher than in IC bone digests (20-fold higher for both, *p* = 0.0002 and *p* = 0.0483, respectively).

### Confirmation of the CD56+ subset involvement in new bone formation in OA

An apparent increase in CD56+ cells in the subchondral bone remodelling areas, its notable pro-osteogenic gene expression and the presence on some osteoblasts in OA trabecular bone suggested that CD56+ BMSCs could be directly involved in subchondral trabeculae thickening and reorganisation in OA^38^.

**Figure 4:**
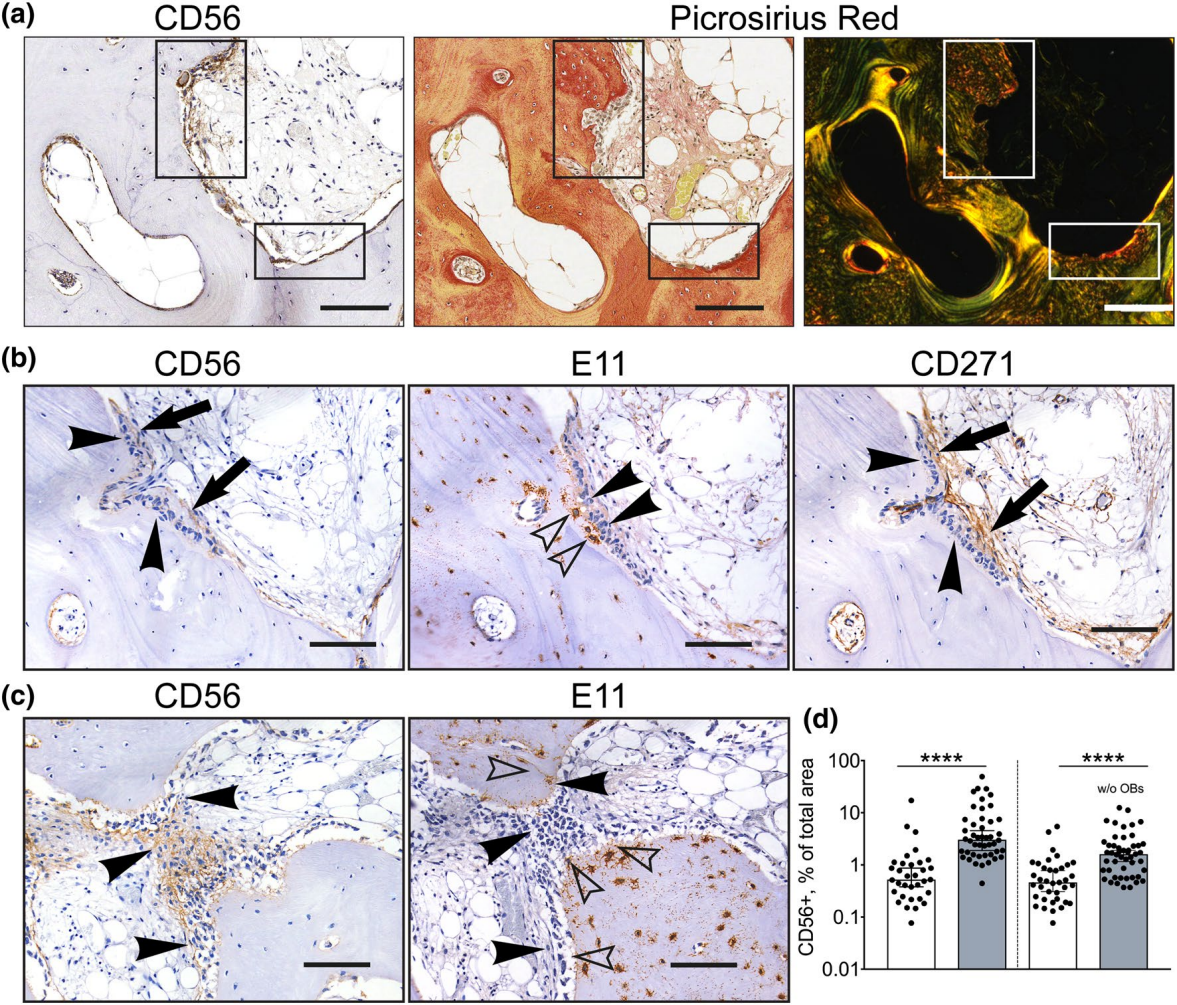
CD56+ subset involvement in new bone formation in OA. (**a**) Co-localisation of CD56+ cells (left panel) with the areas of new bone formation indicated by picrosirius red staining visualised under bright field microscopy (middle panel) and polarised light microscopy (right panel); rectangles: areas of interest for CD56+ cells activity. (**b**) and (**c**) Representative adjacent sections from the areas of bone sclerosis. Co-localisation of CD56+ cells (left panel) with immature E11+ osteocytes (middle panel) in comparison to the whole population of CD271+ BMSCs (right panel); (**b**) sagittal section, (**c**) transverse section (through growing trabeculae). (**d**) Graph showing the area of CD56 positivity as percentage of total cavity area (multiple measurements from n = 4 femoral heads). White box: non-sclerotic areas of femoral heads; grey box: sclerotic areas; w/o OBs: without osteoblasts. **** < 0.0001, Kruskal–Wallis test. Arrows: bone lining location of CD56+ and CD271+ cells, arrowheads: osteoblasts, empty arrowheads: E11+ osteocytes. Scale bars 100 μm.

To address this, OA bone sections were first stained with Picrosirius red (PSR), a dye useful for revealing the collagen organization and fibre orientation in connective tissues (Fig. 4a)^39^. Multiple layers of CD56+ cells were present adjacent to bone areas of bright red collagen staining that also contained high density of embedded osteocytes (Fig. 4a, left and middle panels). Under a polarised light, the differences in collagen fibres’ organisation adjacent to the bone surface and inside the bone became more striking, and the bone areas where CD56+ and recently embedded osteocytes co-localised, were stained red while the remaining bone appeared yellow-to-green, indicating differing degrees of collagen organisation consequent to its maturity.

To investigate whether CD56+ cell accumulation was indeed associated with newly-formed, immature bone tissue (Fig. 4a, right panel), consecutive femoral head OA bone sections were next stained with antibodies against CD271, CD56 and E11, a marker for young, immature osteocytes^40^. The staining revealed the presence of CD56+ cells including some osteoblasts in a direct proximity bone containing high densities of E11+ immature osteocytes (Fig. 4b, left and middle panels). CD271+ cells were more broadly distributed, as expected, but notably excluded osteoblasts (Fig. 4b, right panel). The direct proximity of CD56+ cells and immature osteocytes was even more apparent on a transverse section across the top of the growing trabeculae (Fig. 4c). This staining pattern indicated the osteogenic (i.e. bone-forming) potential of the CD56+ BMSC subset in OA bone.

CD56 staining was next quantified in large sections of femoral heads in relation to the degree of bone sclerosis, as previously described^9^. The areas of interest were traced selecting out the bone and cavity areas followed by calculating the percentage of CD56 positive area in relation to total cavity area (Supplementary Fig. 2). The analysis revealed that the CD56 positive areas were significantly increased in the sclerotic areas of femoral heads (defined as having bone area representing more than 60% of the total area^9^) in comparison to donor matched non-sclerotic areas. Based on median values, the CD56+ cell presence was 5.8-fold higher in the sclerotic areas (*p* < 0.0001) compared to the non-sclerotic areas (Fig. 4d, left panel). When quantification of CD56+ cells was repeated to exclude the osteoblasts, the same trend for higher CD56 positivity in the sclerotic areas (3.5-fold higher, *p* < 0.0001) was evident (Fig. 4d, right panel). This suggests that CD56+ cells actively contributed to bone formation activity in OA.

## Discussion

An increase in the subchondral bone volume is a characteristic feature of late-stage OA^41–43^ and has been previously linked to a preferential BMSC commitment towards osteogenesis in both animals^7^ and humans^9^. The current study has identified a BMSC subset with a characteristic CD271+CD56+ phenotype that directly mediates an enhanced osteogenic activity in OA bone. Whereas previous studies have focussed on the perivascular CD271+CD146+ BMSCs^17^, this is the first study to demonstrate a distinct identity of the CD271+CD56+ BMSC subset in human bone and to directly compare their gene expression with the CD271+CD146+ perivascular BMSCs. Our data suggest that the loss of surface CD146 marks a gradual transition of BMSCs towards osteochondrogenesis, whereas a gain of CD56 marks a further osteogenic commitment step, which ultimately manifests itself as an accumulation of osteoblasts and immature osteocytes in CD56 cell-rich areas of OA bone. In a recent study, magnetically-sorted CD146+ cells from the rat growth plate exhibited high chondrogenic differentiation capacity in vitro, compared to CD105+ cells^44^. Their seemingly different results to our work could be explained by studying different species (human versus rat), tissue sources (trabecular bone versus the growth plate) and control subpopulations (CD56+ versus CD105+).

In the present study, the multipotential nature of the CD56+ BMSC subset was demonstrated following stringent FACS-based purification followed by in vitro tri-lineage differentiation assays. Their remarkable osteogenic commitment, in their native state, was studied without any culture-expansion and was evidenced by the increased expression of a vast array of bone formation associated genes including *SPP1* (osteopontin) and *BGLAP* (osteocalcin), two major components of bone extracellular matrix^30^, and *OMD* (osteomodulin), a proteoglycan previously proposed as an organiser of bone mineral formation^45^ as well as regulator of type I collagen fibril diameter^46^. Compared to CD146+ BMSCs, CD56+ BMSCs also expressed higher levels of *SOX9*, a chief chondrogenic transcription factor, as well *COMP* (cartilage oligomeric matrix protein), however, the fold-differences were not as striking as those noted for the osteogenic molecules. On the other hand, adipogenesis- and stromal support related molecules were lower expressed in the CD56+ BMSCs, likely due to their downregulation as a result of the CD56+ cell osteogenic commitment. This provided the first demonstration of the intrinsic differences in the lineage commitment between the two phenotypically-distinct BMSC subsets in human bone. In vitro differentiation assays that we have used are however prone to artifact and the gold standard in vivo transplantation of these cells would be required to unequivocally document their multipotency^17,29^.

Previous studies have reported on the existence of CD56+ and CD146+ BMSC subsets in human bone marrow^17,21,47^, but the combined use of these markers has never been used before. In this study, their combined use on bone tissue digests facilitated the identification of a third, double-negative (DN) CD271+ BMSC subset in human bone, which was characterised by the higher-level CXCL12 transcript expression^48^. This DN subset was not specific to OA bone and was also present in both IC and NFF bone but at a lower frequency. Its native gene expression signature suggested its transitional ‘osteochondral’ nature, with a lower adipogenic transcript expression compared to the CD271+CD146+ subset, and lower osteogenic transcript expression compared to the CD271+CD56+ subset. Therefore, it may represent a human equivalent to ‘osteoblast/chondrocyte progenitors’ recently identified in mouse bone marrow using a single-cell RNA sequencing technology^11^. Given the major role of CXCL12 in cell migration^35,49,50^, this DN BMSC subset may facilitate the migration and re-location of BMSCs from blood vessels to the bone formation or remodelling areas, a process believed to be exaggerated in OA-affected bone^51^.

In our recent study, we used BMSC culture-expansion to demonstrate that cell isolates with a higher percentage of CD271+CD56+ BMSCs, obtained after rasping of the femoral canal of OA patients, produced more chondrogenic BMSC cultures compared to donor-matched IC aspirates^22^. In contrast to the present work, the DN subset was not quantified in our previous study, but given its proportion, could have also contributed to the high levels of chondrogenesis observed in these isolates. In accumulation, both sets of data on CD271+CD56+ BMSCs indicate their BMSC nature, a high state of ‘readiness’ for osteochondral differentiation, and their further commitment to osteogenesis in bone affected by OA.

The fact that CD56+ BMSCs appear to directly contribute to bone formation in OA-affected bone was next documented in this study using histology and immunohistochemistry. In control non-OA bone, as well as in OA femoral head areas distant to cartilage damage, the CD271+CD56+ BMSCs were restricted to the bone lining region, while the remaining CD271+ BMSCs located within the stromal architecture and around the blood vessels. In OA-affected bone though, characterised by the active remodelling signs and significantly thickened trabeculae^9^, CD56+ cells were additionally increased in the vicinity of osteoblasts, osteocytes and immature collagen fibrils. Furthermore, osteocytes neighbouring these CD56+ cells were also immature as shown by their morphology and the expression of an early-osteocyte marker E11^9^. CD56 expression was also maintained in some neighbouring multi-layered stromal cells believed to be pre-osteoblasts as well as cuboidal osteoblasts, which were notably negative for CD271. This supported early studies showing that CD56 could be a transient molecule during osteoblast differentiation in skeletal morphogenesis^24^, and indicating their in situ osteogenic commitment^23,52^.

The current evidence on the origin of osteoblasts remains unclear. While some studies indicate that quiescent bone lining cells represent a major source of osteoblasts during adulthood^53–55^, others propose the recruitment/migration of osteoprogenitors from ‘canopy cells’ reported to cover most bone remodelling sites, or from nearby blood vessels^53,54^. Data presented in this study demonstrate the presence of CD271 and CD56 on both the quiescent bone surfaces (in non-OA bone) and on active bone surfaces (in OA-affected bone). Given that these CD56+ cells were found to be highly osteogenically-committed, our study indicates that a remote recruitment of osteoprogenitors from blood vessels to form new osteoblasts in OA bone may not be necessary. The triggers of CD56+ bone lining cell activation in OA may be linked to mechanical loading and osteoclast-induced release of excess TGF-β from the extracellular matrix^7^, and remain to be further elucidated. This knowledge could help to develop new therapeutic strategies for the activation of these CD56+ bone-lining cells in osteoporosis and other diseases of bone loss.

This study is limited by the lack of age-matching between OA and IC bone donors and by low numbers of CD271-positive BMSCs that could be obtained from non-OA trabecular bone (both IC and NFF). Compared to OA bone, all BMSC subsets were considerably reduced in numbers in control non-OA bone, which precluded their gene expression analysis. A more sensitive and specific approach, such as a single-cell RNA sequencing^11^, would be necessary to obtain a gene expression profile of these subsets from their healthy environment, as well as other bone-associated pathologies such as osteoporosis. Additionally, new three-dimensional assays for testing BMSC subsets’ functionality such as a mesosphere assay^29^, would need to be standardised and validated. Mesosphere assay is useful not only because it can serially measure the clonogenic cell content of the sorted population in vitro, but also because these spheres can be subcutaneously implanted into immunodeficient mice to measure in vivo self-renewal of these clonogenic cells^29^. As seen in this study, as well as many other studies^22,26,28,56,57^, standard BMSC culture-expansion leads to significant gene and surface marker expression changes in BMSC subsets and hence there is a growing need for other methods to study native BMSC functional properties and differentiation potentials. It will be very valuable to assess colony forming efficiency of the three different BMSC subsets at clonal density before and after FACS, as the latter can cause a significant loss of BMSCs, and these losses may be unequal across the three subsets. Stable labelling of the subsets with different chromophores and looking at the percentages of chromophore-positive colonies out of total colonies, as in lineage-tracking experiments in animal models^58–60^, would be needed to establish their colony-forming efficiency. Ultimately, the in vivo transplantation into an appropriate tissue environment would be required to assess the clonogenicity and lineage commitment of the sorted cell subsets in their more natural states^17,60–63^.

In summary, this is the first study where the CD271+ BMSCs were dissected into different subsets and compared between each other in OA bone, revealing their distinct phenotypes and gene expression signatures in their native states and avoiding in vitro manipulations that lead to significant changes in gene expression. Identifying and characterising CD271+CD56+ osteoprogenitor cell subset in OA bone provides novel insights into the OA bone remodelling process and potential novel approaches for bone manipulation in OA therapy development^64^. For example, the bone-lining location of CD271+CD56+ BMSCs may facilitate the development of novel biologics and small molecules allowing to target these cells similarly to bisphosphonates in order to re-establish the normal bone turnover. A better knowledge on BMSC subsets in bone could be also useful for new therapy development in diseases associated with an abnormal bone-forming phenotype, such as spondyloarthropathies, or conditions where bone formation is lacking, such as osteoporosis.

## Methods

### Patient samples and bone tissue processing

All patients gave informed consent and this study was undertaken after approval from the Leeds East and Leeds West Research Ethics Committees as well as local National Health Service Research and Development in compliance with the Helsinki Declaration of ethical principles for medical research involving human subjects. For this study a total of N = 34 OA donors (15 males and 19 females) with age range between 40 and 90 years (median = 71) were recruited while controls comprised of healthy iliac crest (IC) bone (N = 11, 6 males and 5 females) with age range between 38 and 78 years (median = 53) and femoral heads from neck of femur fracture (NFF) patients (N = 7, all females) with age range between 67 and 93 years (median = 75). No statistical significance in donor age was found between OA and NFF groups in contrast to IC donors which were significantly younger (Kruskal–Wallis test).

From all bone samples, BMSCs were extracted by enzymatic digestion and cell sorting as described previously^9^. Briefly, trabecular bone was first manually segregated from cortical bone, periosteum/endosteum and cartilage using sterile surgical instruments (scalpel and rongeur) (Supplementary Fig. 3). The trabecular bone samples were then broken down into small fragments (< 1 g) using a 22 cm Stille-Luer bone rongeur and extensively washed with phosphate buffered saline (PBS, Sigma, UK) to remove fat and haematopoietic cells. Trabecular bone fragments were then subjected to 4 h’ incubation at 37 °C with 0.22% collagenase (Worthington Biochemical, USA) (corresponding to 3000 U/g of bone). Following digestion, the collagenase solution was separated from the remaining bone by filtering through a 70 µm cell strainer (BD Biosciences, USA), while the bone fragments were washed twice in large volumes of PBS to remove any residual cells. These were pooled with the original collagenase solution, centrifuged (300xg for 5 min) and resuspended in DMEM (Thermo Fisher, USA) (commonly 15–30 ml), containing 10% foetal calf serum (Biosera, France) and 1% penicillin/streptomycin (Thermo Fisher).

### BMSC subset isolation using fluorescence-activated cell sorting (FACS)

To enumerate and isolate native BMSC subsets, up to 10^6^ of the enzymatically released cells were incubated with 15 µl of fluorescein isothiocyanate (FITC)-conjugated CD45 (Agilent Dako, USA), 20 µl allophycocyanin (APC)-conjugated CD271, 20 μl phycoerythrin (PE)-conjugated CD146 (both from Miltenyi Biotec, Germany) and 10 μl of brilliant violet 21 (BV21)-conjugated CD56 (Biolegend, USA) for 20 min at room temperature, washed in FACS buffer and centrifuged (5 min at 300×*g*). Cells were re-suspended in 500 µl FACS buffer, filtered through a 72 μm cell strainer cap (Corning Falcon, USA) into a fresh tube to remove any remaining bone debris or cell clumps. To exclude the dead cells, 10 µl 7-amino-actinomycin D (7-AAD) (Miltenyi Biotec) was added before the cell sorting procedure. Sorting gates were first created by selecting CD45lowCD271+ cells, comprising the BMSC population^6,9^, while the CD45+CD271- gate was used for the collection of haematopoietic lineage cells (HLCs), which were also analysed as control population for BMSCs. The subsequent isolation of BMSCs subsets was based on the gates for CD56 and CD146 positive cells set using the corresponding isotype control antibodies (Fig. 1a). A CD271+ subset negative for both CD146 and CD56, the double-negative (DN) subset, was also collected. In general, an average of 3 × 10^3^ cells (range 0.5 × 10^3^–1.5 × 10^4^ cells) of CD56+ cells, the rarest BMSC subset, were collected using a BD Influx cell sorter (BD Biosciences). Sort-purified BMSC subsets and HLCs were collected directly into 100 μl of RNA lysis buffer (Norgen Biotek, Canada) for downstream gene expression analysis.

### Gene expression

Total RNA from FACS-purified BMSC subsets and control HLCs was extracted using the Single Cell RNA Extraction kit (Norgen Biotek) following manufacturer’s protocol and on-column DNase I treated (Applied Biosystems, USA). Total RNA was then quantified and used to synthesize first-strand cDNA using the High-Capacity cDNA Reverse Transcription Kit (Applied Biosystems) according to manufacturer’s instructions. qPCR was performed on a QuantStudio 7 Flex Real-Time PCR System (Applied Biosystems) using TaqMan Low Density Array (TLDA) cards in 96a format (Thermo Fisher), designed using exon spanning assays wherever possible, the full list of transcripts presented in Supplementary Table 1. Gene expression levels were normalised relative to housekeeping gene *HPRT1* then calculated using the 2^−ΔCt^ method.

Gene expression analysis for major lineage transcripts (*RUNX2* and *BGLAP* for osteogenesis, *PPARG* and *FABP4* for adipogenesis and *SOX9* and *COL2A1* for chondrogenesis) was also performed on Day 21 differentiated cells from N = 3 OA donors following differentiation induction, as previously described^37,65^.

### Cell motility and differentiation assays

Following cell sorting, the BMSC subsets were placed in culture in StemMACS media for subsequent expansion and in vitro functional assays. For morphological assessment, images of individual cells from each subset were taken at 24 h after sorting and cell circularity was calculated using ImageJ software (version 1.8.0_172 National Institutes of Health, USA available at https://imagej.nih.gov/ij/) using the formula for circularity (4π(area/perimeter^2^) where 0 represented a straight line and 1 represented a perfect circle^36^. Separate experiments were then performed for cell motility analysis. For this, freshly-sorted BMSC subsets from N = 4 OA donors were seeded in a 24 well Ibidi culture plates (Ibidi, Germany) at the seeding density of 1 × 10^4^ cells/cm^2^ in 2 ml of StemMACS media and allowed to expand over one week. The cell attachment, spreading and movement were then tracked using Holomonitor M4 microscope (Phase Holographic Imaging AB, Sweden). After automatic calibration of the background and microscope objective, one field of each sample was focused on by the digital autofocus feature. The cells were then imaged with photo captures every 30 min for 24 h. For the evaluation, at each time-lapse sequence, 25 cells were identified and selected by minimum error histogram-based threshold algorithm of the software (HoloStudio M4 version 2.6.2 available at https://phiab.com/product/holomonitor-app-suite-software). By tracking the cells, the motility of each subset was automatically analysed over time, as previously described^66^. Genes’ selections were based on our previous studies^66–68^ as well as other studies^69^.

To assess multipotentiality of the sorted subsets, freshly-sorted BMSC subsets (N = 3 donors) were firstly seeded in 25 cm^2^ culture flasks (Corning) and expanded in StemMACS media for one passage to generate sufficient numbers of BMSCs for in vitro differentiation assays. Differentiation was then performed by culturing matched BMSC subsets in osteogenic, adipogenic or chondrogenic media (all from Miltenyi Biotec), with media changed twice a week, and analysed on day 21, as previously described^37,65^.

### Histology and immunohistochemistry

Trabecular bone samples from at least 3 donors were fixed in 4% paraformaldehyde in a 0.1 M phosphate buffer (pH 7.4) and subsequently decalcified in EDTA (Sigma, 0.5 M, pH 7.4) at 4 °C. Thereafter, bone tissue was fixed for another 24 h in buffered formalin solution and mounted in paraffin blocks for picrosirius red staining and immunohistochemistry (n = 3 sections for each stain from each donor).

Immunohistochemistry was employed to evaluate the topography of CD56+ cells in relation to CD271+ BMSCs and E11+ immature osteocytes in osteoarthritic and control bone. Decalcified trabecular bone sections of 5 μm were deparaffinized in xylene and rehydrated through graded ethanol series. The staining was performed using the EnVision+ Dual Link System-HRP (DAB+) (Dako Agilent) kit as previously described^9^. Primary antibodies included: CD271 (1:100 dilution, clone NGFR5, Abcam, Cambridge, UK), CD56 (1:300 dilution, clone EPR2566, Abcam) and E11 (1:200 dilution, clone NZ1, Merck Millipore, USA). The optimal concentration of primary antibodies had been determined in dilution series on test tissue sections (with and without additional blocking with casein) and no primary antibody was used as negative control. Non-specific binding of primary antibodies was additionally controlled by comparison to a different-specificity antibody of the same species origin and isotype, for example anti-Cathepsin K was used as a control for CD56 (both are rabbit polyclonal IgG). Slides were scanned on Leica Aperio AT2 and images captured using Aperio Imagescope as previously described^9^. Quantification was performed using Nuance Multispectral Imaging System (Calliper Life Sciences, USA) as percentage of positively stained area within a defined trabecular bone cavity area and region of interest, using a minimum of 10 regions per patient as described previously^9^.

Immunofluorescence double staining was performed to detect the BMSCs double-positive for CD271 and CD56 in OA femoral head bone. The tissue was permeabilised to allow intracellular staining with 0.25% Triton-X-100 (Sigma) for 15 min at room temperature and blocked using 5% Casein solution (Sigma) for 30 min at room temperature before 1 h incubation with the same primary antibodies for CD271 and CD56 used for IHC. After 1-h incubation at room temperature, the primary antibodies were washed in Tris buffered saline (Sigma) for 5 min and the appropriate conjugated secondary antibodies were added to the tissue sections for 1 h at room temperature: Alexa Fluor 488 (1:200, Thermo Fisher) for CD271 and Alexa Fluor 647 (1:200, Abcam) for CD56. After washing, the slides were mounted with a coverslip using VectaShield with DAPI (Vector Laboratories, USA). Fluorescence was analysed using a Nikon A1 confocal laser microscope (Nikon) and multiple fluorescent images were acquired and presented using NIS Elements software (Nikon). No primary antibody slide was used as control stained with both secondary antibodies in order to set the threshold and normalise for background signal.

Picrosirius red was used to stain and visualise the collagen fibres in OA femoral heads (n = 4) in order to indicate areas of new bone formation. Following dewaxing and rehydration, the slides were incubated for 1 h at room temperature in 0.1% PSR solution (Sigma). Next, the slides were briefly washed for 1–2 s in 0.5% acetic acid before being mounted with DPX (Leica, UK). The slides were visualised using brightfield as well as polarized light microscopy (Nikon) in order to evaluate the size and organization of collagen fibrils of subchondral bone in parallel with adjacent slides stained for CD56.

### Statistics

The statistical analyses were performed using Friedman test for the donor matched CD271+ BMSC subsets (CD56+, DN and CD146+) while Kruskal–Wallis analysis was used for the intergroup differences between unmatched data sets, both corrected with the Bonferroni–Dunn multiple-group comparison. Mann–Whitney test was used for comparisons between two groups. Results are presented as scatter dots plots with medians or box and whisker plots, with boxes representing the upper and lower quartiles of the median, and whiskers representing the interquartile range, calculated using GraphPad Prism version 7 software. Results were considered significantly different at p < 0.05 (with significance level denoted as *p < 0.05, **p < 0.01, ***p < 0.001 and ****p < 0.001).

## Supplementary information

Supplementary information.

## References

[1] Lories RJ, Luyten FP (2011). The bone–cartilage unit in osteoarthritis. Nat. Rev. Rheumatol..

[2] Goldring SR, Goldring MB (2016). Changes in the osteochondral unit during osteoarthritis: structure, function and cartilage-bone crosstalk. Nat. Rev. Rheumatol..

[3] Henrotin Y, Pesesse L, Sanchez C (2012). Subchondral bone and osteoarthritis: biological and cellular aspects. Osteoporos. Int..

[4] Barry F, Murphy M (2013). Mesenchymal stem cells in joint disease and repair. Nat. Rev. Rheumatol..

[5] McGonagle D, Baboolal TG, Jones E (2017). Native joint-resident mesenchymal stem cells for cartilage repair in osteoarthritis. Nat. Rev. Rheumatol..

[6] Jones E (2010). Large-scale extraction and characterization of CD271+ multipotential stromal cells from trabecular bone in health and osteoarthritis: implications for bone regeneration strategies based on uncultured or minimally cultured multipotential stromal cells. Arthritis Rheum..

[7] Zhen G (2013). Inhibition of TGF-β signaling in mesenchymal stem cells of subchondral bone attenuates osteoarthritis. Nat. Med..

[8] Campbell TM (2016). Mesenchymal stem cell alterations in bone marrow lesions in hip osteoarthritis. Arthritis Rheumatol..

[9] Ilas DC (2019). The simultaneous analysis of mesenchymal stem cells and early osteocytes accumulation in osteoarthritic femoral head sclerotic bone. Rheumatol. (Oxf.).

[10] James S (2015). Multiparameter analysis of human bone marrow stromal cells identifies distinct immunomodulatory and differentiation-competent subtypes. Stem Cell Rep..

[11] Wolock SL (2019). Mapping distinct bone marrow niche populations and their differentiation paths. Cell Rep..

[12] Harichandan A, Bühring HJ (2011). Prospective isolation of human MSC. Best Pract. Res. Clin. Haematol..

[13] Jones E, Schäfer R (2015). Where is the common ground between bone marrow mesenchymal stem/stromal cells from different donors and species?. Stem Cell Res. Ther..

[14] Li H, Ghazanfari R, Zacharaki D, Lim HC, Scheding S (2016). Isolation and characterization of primary bone marrow mesenchymal stromal cells. Ann. N. Y. Acad. Sci..

[15] Kuçi S (2019). Molecular signature of human bone marrow-derived mesenchymal stromal cell subsets. Sci. Rep..

[16] Martel-Pelletier J (2016). Osteoarthritis. Nat. Rev. Dis. Prim..

[17] Tormin A (2011). CD146 expression on primary nonhematopoietic bone marrow stem cells is correlated with in situ localization. Blood.

[18] François S (2006). Local irradiation not only induces homing of human mesenchymal stem cells at exposed sites but promotes their widespread engraftment to multiple organs: a study of their quantitative distribution after irradiation damage. Stem Cells.

[19] Sacchetti B (2007). Self-renewing osteoprogenitors in bone marrow sinusoids can organize a hematopoietic microenvironment. Cell.

[20] Bühring H-J (2009). Phenotypic characterization of distinct human bone marrow-derived MSC subsets. Ann. N. Y. Acad. Sci..

[21] Battula VL (2009). Isolation of functionally distinct mesenchymal stem cell subsets using antibodies against CD56, CD271, and mesenchymal stem cell antigen-1. Haematologica.

[22] Sivasubramaniyan K (2018). Bone marrow-harvesting technique influences functional heterogeneity of mesenchymal stem/stromal cells and cartilage regeneration. Am. J. Sports Med..

[23] Andersen TL (2009). A physical mechanism for coupling bone resorption and formation in adult human bone. Am. J. Pathol..

[24] Lee Y-S, Chuong C-M (2009). Adhesion molecules in skeletogenesis: I. Transient expression of neural cell adhesion molecules (NCAM) in osteoblasts during endochondral and intramembranous ossification. J. Bone Miner. Res..

[25] Churchman SM (2013). Yield optimisation and molecular characterisation of uncultured CD271+ mesenchymal stem cells in the reamer irrigator aspirator waste bag. Eur. Cell Mater..

[26] Churchman SM (2012). Transcriptional profile of native CD271+ multipotential stromal cells: evidence for multiple fates, with prominent osteogenic and WNT pathway signaling activity. Arthritis Rheum..

[27] Ganguly P (2019). The analysis of in vivo aging in human bone marrow mesenchymal stromal cells using colony-forming unit-fibroblast assay and the CD45lowCD271+ phenotype. Stem Cells Int..

[28] Qian H, Le Blanc K, Sigvardsson M (2012). Primary mesenchymal stem and progenitor cells from bone marrow lack expression of CD44 protein. J. Biol. Chem..

[29] Ghazanfari R, Li H, Zacharaki D, Lim HC, Scheding S (2016). Human non-hematopoietic CD271pos/CD140alow/neg bone marrow stroma cells fulfill stringent stem cell criteria in serial transplantations. Stem Cells Dev..

[30] Wei J, Karsenty G (2015). An overview of the metabolic functions of osteocalcin. Rev. Endocr. Metab. Disord..

[31] Hessle L (2013). The skeletal phenotype of chondroadherin deficient mice. PLoS ONE.

[32] Rehn AP, Cerny R, Sugars RV, Kaukua N, Wendel M (2008). Osteoadherin is upregulated by mature osteoblasts and enhances their in vitro differentiation and mineralization. Calcif. Tissue Int..

[33] Tsai F-Y (1994). An early haematopoietic defect in mice lacking the transcription factor GATA-2. Nature.

[34] Li X, Huynh H, Zuo H, Salminen M, Wan Y (2016). Gata2 is a rheostat for mesenchymal stem cell fate in male mice. Endocrinology.

[35] Greenbaum A (2013). CXCL12 in early mesenchymal progenitors is required for haematopoietic stem-cell maintenance. Nature.

[36] Müller S (2019). Osteogenic potential of heterogeneous and CD271-enriched mesenchymal stromal cells cultured on apatite-wollastonite 3D scaffolds. BMC Biomed. Eng..

[37] Fragkakis EM (2018). Vertebral body versus iliac crest bone marrow as a source of multipotential stromal cells: comparison of processing techniques, tri-lineage differentiation and application on a scaffold for spine fusion. PLoS ONE.

[38] Chen Y (2018). Subchondral trabecular rod loss and plate thickening in the development of osteoarthritis. J. Bone Miner. Res..

[39] Lattouf R (2014). Picrosirius red staining: a useful tool to appraise collagen networks in normal and pathological tissues. J. Histochem. Cytochem..

[40] Zhang K (2006). E11/gp38 selective expression in osteocytes: regulation by mechanical strain and role in dendrite elongation. Mol. Cell. Biol..

[41] Suri S, Walsh DA (2012). Osteochondral alterations in osteoarthritis. Bone.

[42] Burr DB, Gallant MA (2012). Bone remodelling in osteoarthritis. Nat. Rev. Rheumatol..

[43] Lajeunesse D, Massicotte F, Pelletier J-P, Martel-Pelletier J (2003). Subchondral bone sclerosis in osteoarthritis: not just an innocent bystander. Mod. Rheumatol..

[44] Wu YX (2017). CD146+ skeletal stem cells from growth plate exhibit specific chondrogenic differentiation capacity in vitro. Mol. Med. Rep..

[45] Sommarin Y, Wendel M, Shen Z, Hellman U, Heinegârd D (1998). Osteoadherin, a cell-binding keratan sulfate proteoglycan in bone, belongs to the family of leucine-rich repeat proteins of the extracellular matrix. J. Biol. Chem..

[46] Tashima T, Nagatoishi S, Sagara H, Ohnuma S, Tsumoto K (2015). Osteomodulin regulates diameter and alters shape of collagen fibrils. Biochem. Biophys. Res. Commun..

[47] Bühring H-J (2007). Novel markers for the prospective isolation of human MSC. Ann. N. Y. Acad. Sci..

[48] Kunisaki Y, Birbrair A (2019). Pericytes in bone marrow. Advances in Experimental Medicine and Biology.

[49] Sugiyama T, Kohara H, Noda M, Nagasawa T (2006). Maintenance of the hematopoietic stem cell pool by CXCL12-CXCR4 chemokine signaling in bone marrow stromal cell niches. Immunity.

[50] Kitaori T (2009). Stromal cell-derived factor 1/CXCR4 signaling is critical for the recruitment of mesenchymal stem cells to the fracture site during skeletal repair in a mouse model. Arthritis Rheum..

[51] Qin HJ (2019). SDF-1/CXCR4 axis coordinates crosstalk between subchondral bone and articular cartilage in osteoarthritis pathogenesis. Bone.

[52] Kristensen HB, Andersen TL, Marcussen N, Rolighed L, Delaisse J-M (2013). Increased presence of capillaries next to remodeling sites in adult human cancellous bone. J. Bone Miner. Res..

[53] Matic I (2016). Quiescent bone lining cells are a major source of osteoblasts during adulthood. Stem Cells.

[54] Hauge EM, Qvesel D, Eriksen EF, Mosekilde L, Melsen F (2001). Cancellous bone remodeling occurs in specialized compartments lined by cells expressing osteoblastic markers. J. Bone Miner. Res..

[55] Kristensen HB, Andersen TL, Marcussen N, Rolighed L, Delaisse J-M (2014). Osteoblast recruitment routes in human cancellous bone remodeling. Am. J. Pathol..

[56] Harichandan A, Sivasubramaniyan K, Bühring H-J (2012). Prospective isolation and characterization of human bone marrow-derived MSCs. Adv. Biochem. Eng. Biotechnol..

[57] Tsai TL, Li WJ (2017). Identification of bone marrow-derived soluble factors regulating human mesenchymal stem cells for bone regeneration. Stem Cell Rep..

[58] Tong W (2019). Periarticular mesenchymal progenitors initiate and contribute to secondary ossification center formation during mouse long bone development. Stem Cells.

[59] Ono N, Ono W, Nagasawa T, Kronenberg HM (2014). A subset of chondrogenic cells provides early mesenchymal progenitors in growing bones. Nat. Cell Biol..

[60] Worthley DL (2015). Gremlin 1 identifies a skeletal stem cell with bone, cartilage, and reticular stromal potential. Cell.

[61] Gothard D, Greenhough J, Ralph E, Oreffo ROC (2014). Prospective isolation of human bone marrow stromal cell subsets: a comparative study between Stro-1-, CD146- and CD105-enriched populations. J. Tissue Eng..

[62] Duchamp De Lageneste O (2018). Periosteum contains skeletal stem cells with high bone regenerative potential controlled by Periostin. Nat. Commun..

[63] Chan CKF (2015). Identification and specification of the mouse skeletal stem cell. Cell.

[64] Padilla S, Sánchez M, Orive G, Anitua E (2017). Human-based biological and biomimetic autologous therapies for musculoskeletal tissue regeneration. Trends Biotechnol..

[65] Jones EA (2008). Synovial fluid mesenchymal stem cells in health and early osteoarthritis: detection and functional evaluation at the single-cell level. Arthritis Rheum..

[66] Owston HE (2019). Colony formation, migratory, and differentiation characteristics of multipotential stromal cells (MSCs) from “clinically accessible” human periosteum compared to donor-matched bone marrow MSCs. Stem Cells Int..

[67] Baboolal TG (2014). Intrinsic multipotential mesenchymal stromal cell activity in gelatinous Heberden’s nodes in osteoarthritis at clinical presentation. Arthritis Res. Ther..

[68] Churchman SM, Boxall SA, McGonagle D, Jones EA (2017). Predicting the remaining lifespan and cultivation-related loss of osteogenic capacity of bone marrow multipotential stromal cells applicable across a broad donor age range. Stem Cells Int..

[69] Paic F (2009). Identification of differentially expressed genes between osteoblasts and osteocytes. Bone.

