# Supplementary material for: The osteogenic commitment of CD271+CD56+ bone marrow stromal cells (BMSCs) in osteoarthritic femoral head bone
Source: Sci Rep. 2020 Jul 7;10:11145. doi: 10.1038/s41598-020-67998-0 (PMC7341749; doi:10.1038/s41598-020-67998-0)
Supplement: Supplementary file 1 — Supplementary information. [file 41598_2020_67998_MOESM1_ESM.pdf]

Supplementary Information

The osteogenic commitment of CD271+CD56+ bone marrow stromal cells (BMSCs) in osteoarthritic femoral head bone

Dragos C. Ilas, PhD [1], Thomas G. Baboolal, PhD [1,2], Sarah M. Churchman, PhD [1], William G. Jones, BSc, MSc [1,3], Peter V. Giannoudis, BSc, MB, MD, FACS, FRCS [1], Hans-Jörg Bühring, PhD [4], Dennis McGonagle, PhD, FRCPI [1,2], Elena Jones, PhD [1*]

[1] Leeds Institute of Rheumatic and Musculoskeletal Medicine, The University of Leeds, Leeds, UK

[2] NIHR Leeds Musculoskeletal Biomedical Research Unit, Leeds, United Kingdom.

[3] Institute of Medical and Biological Engineering, University of Leeds, Leeds, UK

[4] Department of Internal Medicine II, University of Tübingen, Germany

***Address of correspondence:**

Dr. Elena Jones
Leeds Institute of Rheumatic and Musculoskeletal Medicine
Room 5.23 Clinical Sciences Building
St James's University Hospital
Leeds LS9 7TF
Tel: 44 113 2065647
FAX: 44 113 3438502
e.jones@leeds.ac.uk

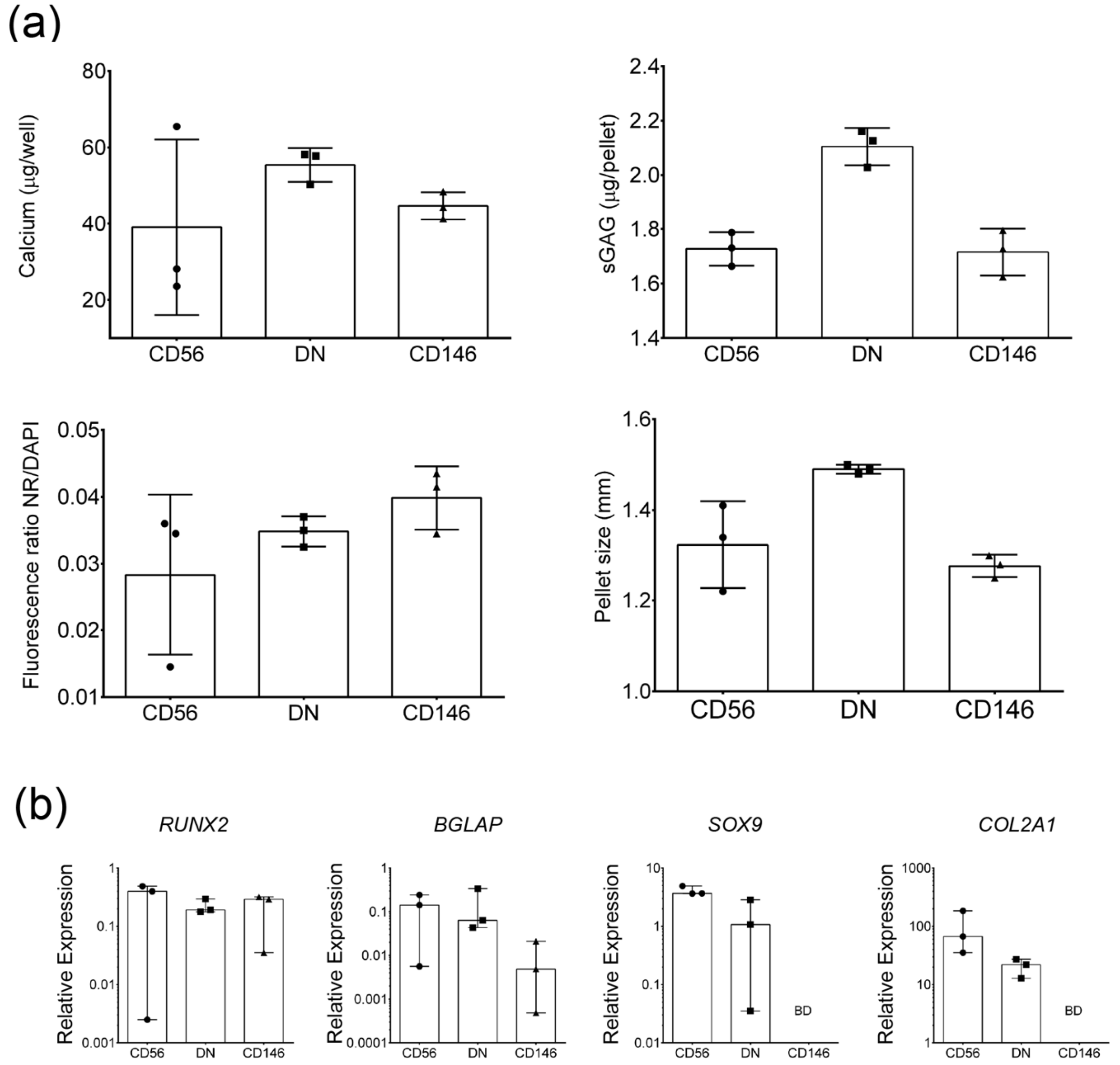

Supplementary Figure 1. Quantitative assessment of tri-lineage multipotentiality of CD271+ BMSCs subsets following minimal culture-expansion.

(a): Functional data. Calcium production of sorted, culture-expanded and osteogenically-induced cells following 21 days of induction (top left). Adipogenesis quantification by uptake of Nile red normalised to DAPI following 21 days of adipogenic induction (bottom left). Quantification of GAG in chondrogenic pellets (top right) and measurement of pellet sizes (bottom right). Functional assays data is presented as dot plots with bars indicating mean values and whiskers representing standard deviation, n=3 donors. (b) Gene expression data. Transcriptional analysis was performed for osteogenic transcripts *RUNX2* and *BGLAP* and chondrogenic transcripts *SOX9* and *COL2A1* on day 21 post-induction and data are presented as dot plots with bars indicating median values and whiskers representing interquartile ranges; n=3 donors. BD: below detection levels.

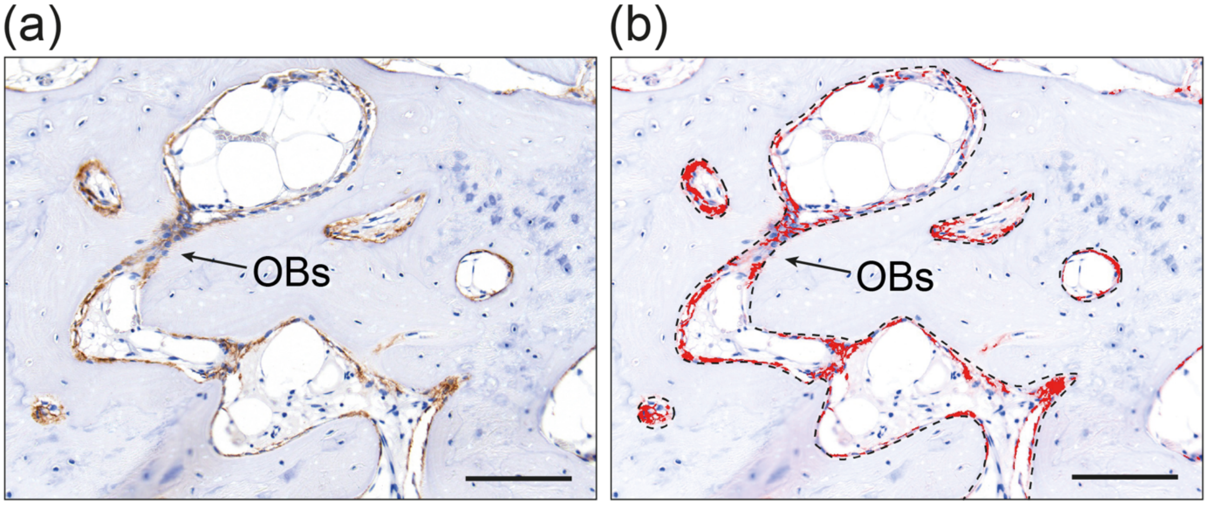

Supplementary Figure 2. Quantification of CD56+ cells in OA bone.

Representative photomicrographs showing IHC of CD56 positive cells in OA femoral heads sclerotic areas (a). The areas were selected for quantification using Nuance software by tracing a dotted line around the bone cavity areas (b). CD56 area quantification was performed based on DAB+ staining (brown), within the selected bone cavity areas (dashed lines) above negative control threshold (indicated by red colour). OBs: osteoblasts; Scale bar: 100 µm.

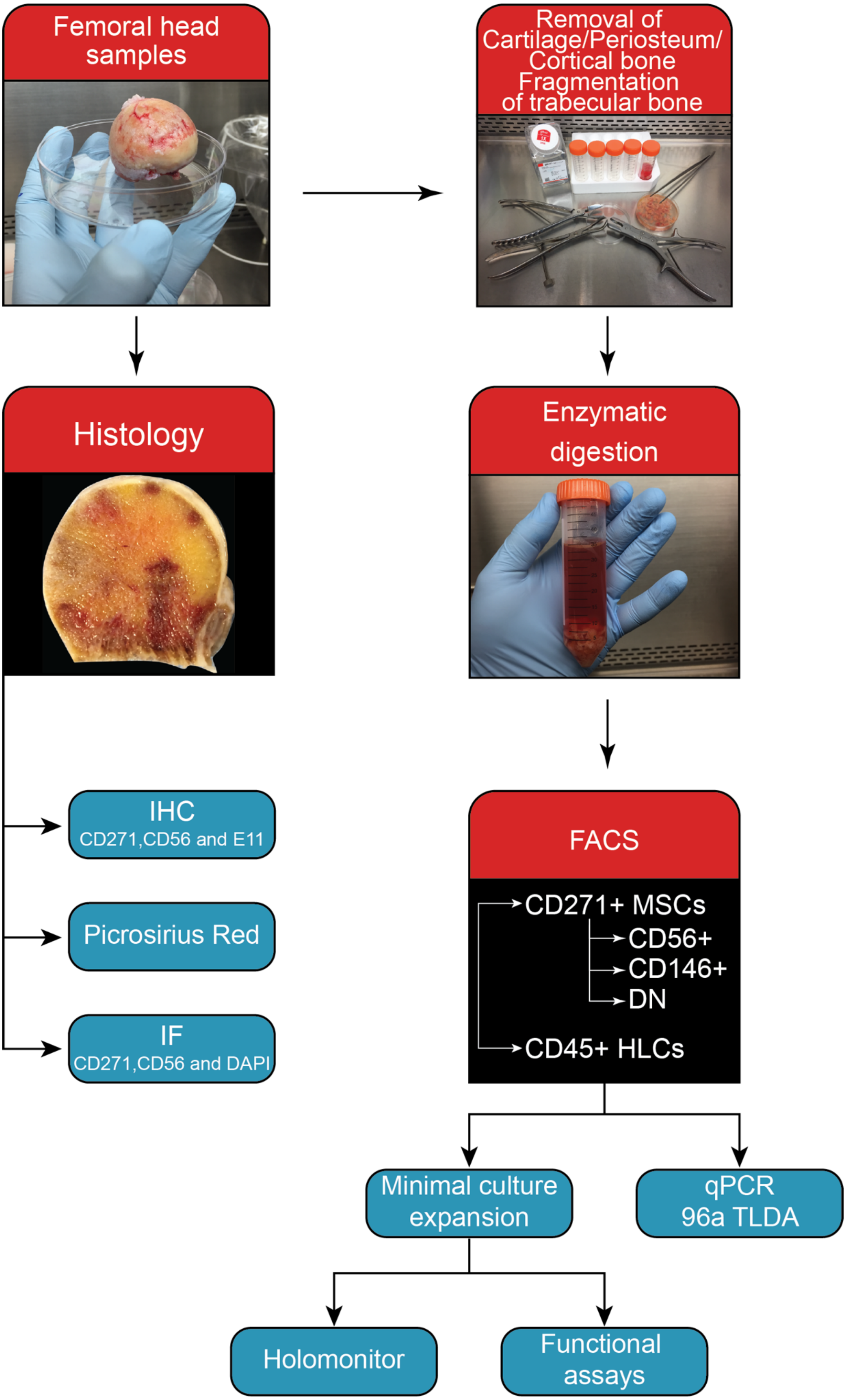

Femoral head samples
Removal of Cartilage/Periosteum/ Cortical bone Fragmentation of trabecular bone
Histology
Enzymatic digestion
IHC
CD271,CD56 and E11
Picrosirius Red
IF
CD271,CD56 and DAPI
FACS
CD271+ MSCs
CD56+
CD146+
DN
CD45+ HLCs
Minimal culture expansion
qPCR
96a TLDA
Holomonitor
Functional assays

Supplementary Figure 3. Experimental design for CD271+ BMSCs subsets isolation and characterisation from OA femoral head trabecular bone samples.

For histological analysis, femoral heads from n=4 OA donors were slowly decalcified in EDTA solution, bisected using a standard pathology knife and tissue sections were used for IHC, IF or Picrosirius Red staining. To obtain trabecular bone-resident live cells, soft tissue (ligament, periosteum) and cartilage were first removed from femoral heads using a scalpel then cortical bone was taken out using rongeur. The remaining trabecular bone was then mechanically broken down into small fragments using rongeur and enzymatically digested. From the obtained cell suspension, the BMSCs were isolated by FACS and used for subsequent gene expression analysis and functional in vitro assays. For gene expression analysis a total of n=6 OA donors were used for analysis by qPCR using a 96a gene panel TaqMan low-density array card. Each of the three subsets (CD56+, CD146+ and DN) isolated by FACS from n=3 OA donors were induced towards osteogenic, adipogenic and chondrogenic lineages and assessed by standard in-vitro quantitative and qualitative assays. The three subsets from different n=3 OA donors were used to study cell motility using the holographic imaging (Holomonitor)

Supplementary Table 1. List of TaqMan assays ID and the corresponding gene names used for gene expression analysis.

Assay ID	Gene Symbol	Synonyms	References
Hs00153936_m1	*ACAN*	Aggrecan	[67]
Hs00909449_m1	*ACTA2*	actin, alpha 2, smooth muscle, aorta	[26,67,68]
Hs00244715_m1	*ACVR1B*	activin A receptor, type IB	[26,67,68]
Hs00155658_m1	*ACVR2A*	activin A receptor, type IIA	[26,67,68]
Hs00758162_m1	*ALPL*	alkaline phosphatase, liver/bone/kidney	[17,29,67,69]
Hs00181613_m1	*ANGPT1*	angiopoietin 1	[29,67]
Hs01101127_m1	*ANGPTL4*	angiopoietin-like 4	[28,67]
Hs03044164_m1	*BAMBI*	BMP and activin membrane-bound inhibitor homolog	[26,67,68]
Hs00609452_g1	*BGLAP*	bone gamma-carboxyglutamate protein (osteocalcin)	[26,67–69]
Hs00154192_m1	*BMP2*	bone morphogenetic protein 2	[26,67,68]
Hs00370078_m1	*BMP4*	bone morphogenetic protein 4	[29,67]
Hs00234930_m1	*BMP5*	bone morphogenetic protein 5	[29,67]
Hs00403062_m1	*BMPER*	BMP binding endothelial regulator	[26,67,68]
Hs00831730_s1	*BMPR1A*	bone morphogenetic protein receptor, type IA	[26,67,68]
Hs00156438_m1	*CDH11*	cadherin 11, type 2, OB-cadherin	[29,67,69]
Hs00901463_m1	*CDH5*	cadherin 5, type 2, VE-cadherin	[26,67,68]
Hs00269972_s1	*CEBPA*	CCAAT/enhancer binding protein (C/EBP), alpha	[17,29,67]
Hs00154382_m1	*CHAD*	Chondroadherin	[67]
Hs00253550_m1	*CLEC4M*	CD209 molecule,C-type lectin domain family 4, member M	[26,67,68]
Hs00166657_m1	*COL10A1*	collagen, type X, alpha 1	[67]
Hs01076777_m1	*COL1A1*	collagen, type I, alpha 1	[28,67]
Hs01028971_m1	*COL1A2*	collagen, type I, alpha 2	[28,67]
Hs00264051_m1	*COL2A1*	collagen, type II, alpha 1	[67]
Hs00164359_m1	COMP	Cartilage Oligomeric Matrix Protein	[28,67]

Hs00426981_m1	*CSPG4*	chondroitin sulfate proteoglycan 4	[67]
Hs00171022_m1	*CXCL12*	chemokine (C-X-C motif) ligand 12 (stromal cell-derived factor 1)	[29,67]
Hs00601975_m1	*CXCL2*	C-X-C Motif Chemokine Ligand 2	[26,67,68]
Hs00322497_m1	*DAAM2*	dishevelled associated activator of morphogenesis 2	[26,67,68]
Hs00178815_m1	*DDR2*	discoidin domain receptor tyrosine kinase 2	[26,67,68]
Hs00182901_m1	*DVL2*	dishevelled, dsh homolog 2	[26,67,68]
Hs00358886_m1	*EFNA1*	ephrin-A1	[26,67,68]
Hs00174752_m1	*EPHB4*	EPH receptor B4	[29,67]
Hs00191912_m1	*EPYC*	epiphycan	[67]
Hs00609791_m1	*FABP4*	fatty acid binding protein 4, adipocyte	[29,67]
Hs00170454_m1	*FGF5*	fibroblast growth factor 5	[26,67,68]
Hs00241111_m1	*FGFR1*	fibroblast growth factor receptor 1	[26,67,68]
Hs00179829_m1	*FGFR3*	fibroblast growth factor receptor 3	[26,67,68]
Hs00173503_m1	*FRZB*	frizzled-related protein	[28,29,67]
Hs00268943_s1	*FZD1*	frizzled homolog 1	[29,67]
Hs00201853_m1	*FZD4*	frizzled homolog 4	[29,67]
Hs00275833_s1	*FZD7*	frizzled homolog 7	[29,67]
Hs00259040_s1	*FZD8*	frizzled homolog 8	[26,67,68]
Hs00268954_s1	*FZD9*	frizzled homolog 9	[29,67]
Hs99999905_m1	*GAPDH*	glyceraldehyde-3-phosphate dehydrogenase	[29,67]
Hs00231119_m1	*GATA2*	GATA binding protein 2	[29,67]
Hs00167060_m1	*GDF5*	Growth Differentiation Factor 5	[67]
Hs01075601_m1	*GHR*	growth hormone receptor	[29,67]
Hs00748445_s1	*GJA1*	gap junction protein, alpha 1, 43kDa	[26,67,68]
Hs00157103_m1	*HAPLN1*	Hyaluronan And Proteoglycan Link Protein 1	[67]
Hs99999909_m1	*HPRT1*	hypoxanthine phosphoribosyltransferase 1	[26,67,68]
Hs99999041_m1	*IFNG*	interferon, gamma,	[26,67,68]
Hs00181211_m1	*IGFBP3*	insulin-like growth factor binding protein 3	[29,67]

Hs00961622_m1	*IL10*	interleukin 10	[26,67,68]
Hs00174202_m1	*IL7*	interleukin 7	[29,67]
Hs01070036_m1	*JAG1*	jagged 1	[29,67]
Hs00174492_m1	*LEPR*	leptin receptor	[29,67]
Hs01012571_m1	*LPL*	lipoprotein lipase	[28,29,67]
Hs00174838_m1	*MCAM*	melanoma cell adhesion molecule	[17,28,67]
Hs00427183_m1	*MSX1*	msh homeobox 1	[26,67,68]
Hs02379661_g1	*MT2A*	metallothionein 2A	[26,67,68]
Hs00159522_m1	*MYH9*	myosin, heavy chain 9, non-muscle	[26,67,68]
Hs02387400_g1	*NANOG*	Nanog homeobox	[17,67]
Hs00707120_s1	*NES*	nestin	[29,67]
Hs00182120_m1	*NGFR*	nerve growth factor receptor (TNFR superfamily, member 16)	[17,28,67]
Hs00271352_s1	*NOG*	noggin	[26,67,68]
Hs00192325_m1	*OMD*	osteomodulin	[26,67–69]
Hs00190682_m1	PAPSS2	3'-Phosphoadenosine 5'-Phosphosulfate Synthase 2	[67]
Hs00170179_m1	*PCOLCE*	procollagen C-endopeptidase enhancer	[26,67,68]
Hs00998018_m1	*PDGFRA*	platelet-derived growth factor receptor, alpha polypeptide	[26,67,68]
Hs00185122_m1	*PDGFRL*	platelet-derived growth factor receptor-like	[26,67,68]
Hs01370291_g1	*PHOSPHO1*	phosphatase, orphan 1	[26,67,68]
Hs00999632_g1	*POU5F1*	POU class 5 homeobox 1	[17,67]
Hs01115513_m1	*PPARG*	peroxisome proliferator-activated receptor gamma	[29,67]
Hs00160431_m1	*PRELP*	Proline and Arginine Rich End Leucine Rich Repeat Protein	[28,67]
Hs00231079_m1	*RUNX1*	runt-related transcription factor 1	[26,67,68]
Hs00231692_m1	*RUNX2*	runt-related transcription factor 2	[29,67,69]
Hs00173499_m1	*S1PR1*	Sphingosine-1-Phosphate Receptor 1	[26,67,68]
Hs00610060_m1	*SFRP1*	secreted frizzled-related protein 1	[29,67]
Hs00180066_m1	*SFRP4*	secreted frizzled-related protein 4	[26,67,68]

Hs00361747_m1	*SORT1*	sortilin 1	[26,67,68]
Hs01053049_s1	*SOX2*	SRY (sex determining region Y)-box 2	[17,67]
Hs00165814_m1	*SOX9*	SRY (sex determining region Y)-box 9	[29,67]
Hs00541729_m1	*SP7*	Sp7 transcription factor	[26,67,68]
Hs00277762_m1	*SPARC*	secreted protein, acidic, cysteine-rich (osteonectin)	[29,67,69]
Hs00959010_m1	*SPP1*	secreted phosphoprotein 1 (osteopontin, bone sialoprotein I)	[29,67,69]
Hs00559661_m1	*TGFBR2*	transforming growth factor, beta receptor II (70/80kDa)	[26,67,68]
Hs00234257_m1	*TGFBR3*	transforming growth factor, beta receptor III	[29,67]
Hs01113602_m1	*TNFAIP6*	TNF Alpha Induced Protein 6	[67]
Hs00900360_m1	*TNFRSF11B*	tumor necrosis factor receptor superfamily, member 11b (osteoprotegerin)	[26,67,68]
Hs00361186_m1	*TWIST1*	twist homolog 1	[26,67,68]
Hs00382379_m1	*TWIST2*	twist homolog 2	[26,67,68]
Hs01097550_m1	*UGDH*	UDP-Glucose Dehydrogenase	[67]
Hs00900058_m1	*VEGFA*	vascular endothelial growth factor A	[26,67,68]
Hs01099206_m1	*VEGFC*	vascular endothelial growth factor C	[29,67]
Hs00183662_m1	*WIF1*	WNT inhibitory factor 1	[26,67,68]
